# Nurses’ Pain Knowledge, Attitudes, and Perceived Barriers to Timely Analgesia in a Saudi Tertiary Hospital: A Cross-Sectional Study

**DOI:** 10.3390/nursrep16070245

**Published:** 2026-07-15

**Authors:** Khulud Adel Abudawood, Rawan Almutairi

**Affiliations:** 1 College of Nursing, King Saud bin Abdulaziz University for Health Sciences, Jeddah 21423, Saudi Arabia; 2 King Abdullah International Medical Research Center, Jeddah 21423, Saudi Arabia; 3 Ministry of National Guard Health Affairs, Jeddah 21423, Saudi Arabia

**Keywords:** pain knowledge, NKASRP, nurses, analgesia delays, Saudi Arabia, barriers

## Abstract

Pain is highly prevalent during hospitalization, and inadequate assessment contributes to avoidable suffering, prolonged length of stay, and increased resource use. Nurses play a central role in pain assessment, timely analgesic administration, patient education, and escalation of care. **Background/Objectives:** This study assessed nurses’ knowledge and attitudes regarding pain assessment and management and examined perceived causes of delayed pain treatment and perceived barriers to timely pain control at a tertiary hospital in Saudi Arabia. **Methods:** A descriptive cross-sectional study was conducted in inpatient and outpatient departments at King Abdulaziz Medical City, Jeddah, Saudi Arabia (August–December 2023). Registered nurses providing direct patient care were recruited via convenience sampling. Data were collected using a demographic and clinical practice questionnaire, the Nurses’ Knowledge and Attitudes Survey Regarding Pain (NKASRP), and an adopted instrument assessing perceived causes of delayed pain treatment and perceived barriers. Data were analyzed using descriptive statistics, two-tailed *t*-tests, one-way ANOVA with assumption checks and post hoc testing, Pearson correlations, and multiple linear regression. **Results:** A total of 125 nurses participated; 95.2% were female and 80.8% held a bachelor’s degree. The mean NKASRP composite score was 46.69% (SD = 9.90), indicating a suboptimal knowledge-and-attitudes profile. Age group was significantly associated with NKASRP scores (F = 5.136, *p* = 0.007), and age remained the only significant independent predictor in the multivariable model (b = 0.055, *p* = 0.006). The most frequently perceived delay was contacting a physician for opioid prescription (58.4%), followed by obtaining opioids from the pharmacy (46.4%). Major perceived barriers included insufficient staff–patient communication (60.0%), insufficient patient knowledge of pain control (52.8%), and inadequate staffing (49.6%). **Conclusions:** Nurses demonstrated a low composite knowledge-and-attitudes score and reported communication, workflow, and system-related barriers relevant to timely analgesia. These findings support further prospective evaluation of targeted educational and system-level strategies in similar settings.

## 1. Introduction

Pain remains one of the most common and distressing symptoms experienced by hospitalized patients, and inadequate pain control is associated with delayed recovery, impaired mobility, prolonged hospitalization, emotional distress, and increased healthcare utilization when not adequately managed [1,2]. Acute pain in medical and surgical settings, particularly postoperative pain, requires timely assessment and reassessment because untreated or undertreated pain can intensify physiological stress responses, delay rehabilitation, and diminish patient satisfaction with care [1]. In Saudi Arabia, chronic pain and musculoskeletal conditions continue to impose a substantial burden on the healthcare system, further underscoring the clinical importance of effective pain management [3,4]. These patterns reinforce the clinical importance of ensuring that front-line nurses possess sound pain-related knowledge and can translate that knowledge into timely and appropriate practice.

Nurses play a central role in recognizing pain, performing structured assessments, administering analgesics, evaluating treatment response, educating patients, and escalating concerns within interprofessional care pathways [5,6]. Because pain is fundamentally subjective, accurate management depends not only on documentation but also on the nurse’s ability to interpret patient self-report, behavioral cues, and clinical context accurately [7,8]. However, evidence continues to show important gaps in nurses’ knowledge and attitudes regarding pain assessment and management, including misconceptions about opioid safety, underuse of patient self-report, and difficulty selecting appropriate analgesic routes and reassessment strategies [7,8,9]. In Qatar, Samara et al. reported a mean NKASRP score of only 48% among postoperative nurses, and in Oman, nurses at a national cancer center achieved a mean score of 49.6%, with significant associations between knowledge and education level, clinical experience, and prior pain management training [10,11]. Together, these findings demonstrate that suboptimal pain knowledge and attitudes persist across Gulf settings, despite increasing attention to pain education and quality improvement initiatives.

Pain management is also shaped by workflow and organizational factors. Delays can arise from physician-gated prescribing, pharmacy access processes, staffing constraints, and patient-level reluctance to report pain or accept opioids [12,13]. In Saudi Arabia, nurses have reported barriers related to staffing insufficiency, opioid regulation, and communication limitations, all of which may affect the timeliness of pain relief [13,14]. Although electronic health records can improve standardization and visibility of pain documentation, documentation alone does not ensure better management unless it is linked to effective decision support and workable clinical processes [15,16,17]. Accordingly, the present study did not directly evaluate the functionality of electronic health records (EHRs) or the quality of documentation. Instead, it focused on nurses’ knowledge of pain and attitudes, perceived causes of delay, and perceived barriers within a Saudi tertiary-care context, where nurses are expected to assess pain promptly, communicate effectively with prescribers, and support timely analgesia.

Despite widespread use of NKASRP in pain research, relatively few studies in Saudi Arabia have simultaneously examined nurses’ pain knowledge and attitudes, perceived causes of delayed analgesia, and perceived barriers to pain management within the same tertiary-care setting [9,10,11,13,14]. Clarifying this combination of educational and workflow-related factors is important for identifying targets for future interventions, especially in settings where nurses are expected to promptly recognize pain, coordinate with prescribers, and maintain effective communication with patients and colleagues.

This study aimed to assess nurses’ knowledge and attitudes regarding pain assessment and management and to describe perceived causes of delayed pain treatment and perceived barriers to timely pain control in a tertiary care hospital in Saudi Arabia.

The research questions were as follows:

1. What is the level of nurses’ knowledge and attitudes regarding pain (NKASRP)?
2. Are demographic and professional characteristics associated with NKASRP scores?
3. What are the perceived causes of delays in pain management?
4. What staff, patient, and system-level barriers do nurses report related to pain management?

## 2. Materials and Methods

### 2.1. Study Design and Setting

A descriptive cross-sectional correlational design was used to measure nurses’ pain-related knowledge and attitudes and to explore associations with nurse characteristics. The study was conducted at King Abdulaziz Medical City (KAMC), Jeddah, Saudi Arabia, across inpatient and outpatient departments (critical care, oncology, medical–surgical units, pediatrics, emergency, and outpatient clinics), reflecting the diversity of pain presentations and workflow processes across the institution. Data were collected between August and December 2023.

### 2.2. Participants and Sampling

Eligible participants were registered nurses providing direct patient care and involved in pain assessment and management. Student nurses and nurses primarily in managerial or educational roles were excluded. Convenience sampling was used. An a priori sample size estimate was generated in G*Power 3.1 for a one-way ANOVA with three groups, assuming a medium effect size (f = 0.25), alpha = 0.05, power = 0.80, and k = 3 groups, yielding a required sample of 252. Inflating for an anticipated 15% non-response rate (252 ÷ 0.85 = 296.5, rounded to 297) gave the target recruitment of 297 participants. The achieved sample of 125 registered nurses (response rate ≈ 42.1%) corresponds to a post hoc statistical power of approximately 0.68 for detecting a medium effect in the primary age group comparison; the increased risk of Type II error is discussed in the limitations section.

### 2.3. Instruments

#### 2.3.1. Demographic and Clinical Practice Questionnaire

A structured questionnaire captured demographic variables (age, gender, education level) and professional characteristics (years of experience, clinical area), as well as practice indicators relevant to pain management, including attendance at pain-related education or workshops and self-reported pain documentation practices. Items were developed for this study and aligned with prior surveys assessing pain practices and institutional barriers [6,7,18,19]. Content validity was supported through alignment with prior literature on determinants of pain assessment and documentation practices [5,6,7].

#### 2.3.2. Nurses’ Knowledge and Attitudes Survey Regarding Pain (NKASRP)

Nurses’ knowledge and attitudes were assessed using the Nurses’ Knowledge and Attitudes Survey Regarding Pain (NKASRP), originally developed by Ferrell and McCaffery [9]. The instrument includes 22 true/false items, 17 multiple-choice items, and two case-based scenarios designed to assess pain assessment principles, opioid pharmacology, and pain-related clinical decision-making. Each correct response was scored as 1 and incorrect responses as 0. Missing responses were scored as incorrect in the primary analysis for comparability with prior NKASRP-based reporting. A total score was computed and transformed into a percentage score (0–100%). In this study, reporting thresholds were standardized to 80%, consistent with the conventional NKASRP competency benchmark, and the NKASRP results were interpreted primarily as a composite knowledge-and-attitudes measure rather than as a direct indicator of attitudes alone.

Validity/Reliability: The NKASRP has been widely used internationally and has demonstrated acceptable psychometric performance across multiple studies, supporting its construct validity for assessing pain-related knowledge and attitudes [9,18,20]. In the present sample, the 41-item NKASRP demonstrated acceptable internal consistency (Cronbach’s α = 0.72; computed across all 41 dichotomously scored items from 125 respondents, 5125 item responses). To improve local contextual relevance, the original Percocet reference was replaced with Revacod in one item because Revacod is a commonly available paracetamol/codeine combination in Saudi Arabia. The sterile water injection item reflected the original NKASRP content rather than a local addition. Because minor contextual adaptation was undertaken, sample-specific internal consistency was reported in the present study.

Its continued use in diverse settings, including critical care, oncology, and medical–surgical nursing, supports its utility for identifying knowledge gaps and informing educational priorities [8,18,19].

#### 2.3.3. Perceived Causes of Delay and Barriers to Pain Management

Perceived causes of delayed pain treatment and perceived barriers to pain management were assessed using an instrument adopted from Othman and Al-Atiyyat [12]. The tool covers perceived delay points and barriers across staff-related, patient-related, and healthcare system-related domains. Nurses were permitted to choose more than one applicable response, and the reported percentages therefore represent endorsement frequencies for each listed item rather than mutually exclusive categories.

Validity/reliability: Because this instrument was adopted from previously published work rather than developed for the present study, Face/content validity was supported by grounding the barrier categories in established literature on pain management barriers (knowledge/communication/workflow/system constraints) [11,12]. The original study reported acceptable internal consistency and reliability, with a Cronbach’s coefficient value of 0.771 [12].

### 2.4. Data Collection Procedures

Following ethical and administrative approval, eligible nurses were approached in participating units. The study purpose, anonymity, and voluntary nature were explained, and informed consent was obtained. The questionnaire was administered in English without personal identifiers, since English is the official language for all hospital communication and documentation.

### 2.5. Statistical Analysis

SPSS v25 was used. Descriptive statistics summarized participant characteristics and survey responses. There were no missing responses in the overall NKASRP; however, it was planned to be handled by scoring missing responses as incorrect in the primary analysis. Independent-samples *t*-tests were conducted as two-tailed tests, and one-way ANOVA was used to assess group differences in NKASRP scores. Assumptions for ANOVA were checked using Levene’s test and Shapiro–Wilk tests, and post hoc comparisons were conducted using Tukey and Bonferroni procedures where appropriate. Multiple linear regression was then performed to identify factors independently associated with NKASRP scores. Statistical significance was set at *p* < 0.05.

## 3. Results

### 3.1. Participant Characteristics

The sample (N = 125) was predominantly female (95.2%) and bachelor’s-prepared (80.8%). Mean age was 31.45 years (SD = 8.68). Most participants reported previous pain education (68.8%) and routine pain documentation (97.6%). Because documentation practices were self-reported rather than independently audited, this variable should be interpreted with caution. Participant characteristics are presented in Table 1.

### 3.2. NKASRP Performance (Knowledge and Attitudes)

**Figure 1:**
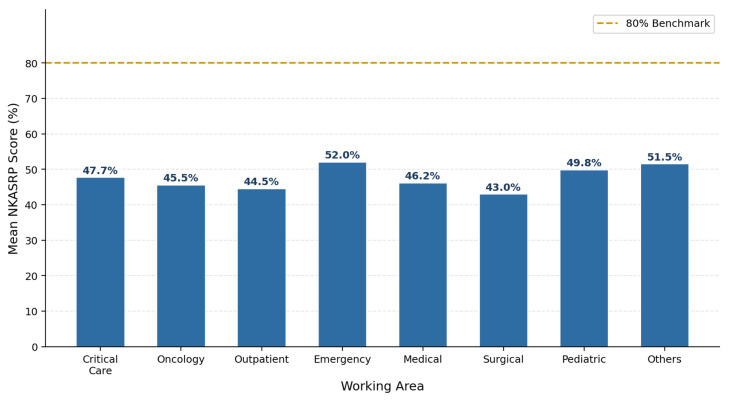
Mean NKASRP score (%) by working area. The dashed line indicates the 80% competency benchmark. Note: The Emergency Department subgroup (n = 3) yields an unstable mean and should be interpreted with caution.

The mean NKASRP composite score was 46.69% (SD = 9.90), well below accepted competency expectations and indicative of a suboptimal overall knowledge-and-attitudes profile. To avoid overinterpreting the composite score as attitudes alone, this study refers to outcomes as a composite knowledge-and-attitudes measure. Item-level performance is presented in Table 2 and Table 3. Mean scores by working area are visualized in Figure 1, although the Emergency Department subgroup should be interpreted cautiously because it only included three participants. Items with high correct-response rates (≥80%) included sedation assessment (81.6%), opioid addiction definition (80.8%), initial opioid dose timing (80.8%), and analgesia meaning (80.0%). Items with the lowest correct-response rates (<10%) were the assessment of a smiling patient (3.2%) and the recommended intramuscular route (5.6%), reflecting specific gaps in clinical reasoning and knowledge of routes of administration.

### 3.3. Association Between NKASRP Scores and Demographic Variables

**Figure 2:**
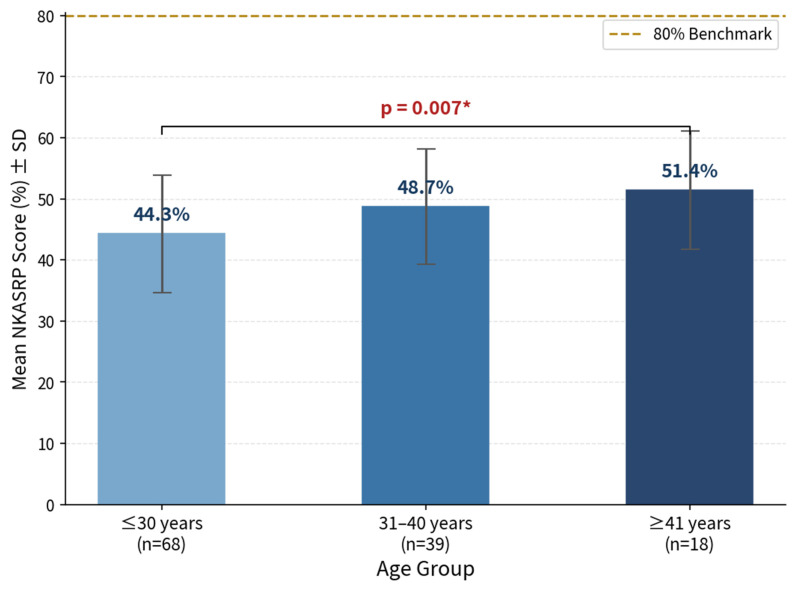
Mean NKASRP score (%) by age group with SD error bars. One-way ANOVA, F = 5.136, * *p* = 0.007. The dashed line indicates the 80% competency benchmark.

Age group was the only variable significantly associated with NKASRP scores in univariate analysis (F = 5.136, *p* = 0.007), with older nurses achieving higher mean scores (Figure 2 and Table 4). No significant associations were identified for gender, years of experience, education level, workshop attendance, documentation practice, or working area. The gender comparison was rechecked using a two-tailed independent-samples *t*-test and was not statistically significant. For the age group ANOVA, assumption checks were acceptable, and post hoc testing indicated that nurses aged ≥41 years scored significantly higher than those aged ≤30 years, whereas the remaining pairwise comparisons were not statistically significant.

In multivariable analysis, age remained the only independent significant predictor of NKASRP score; whereas experience, education, workshop attendance, and working area were not statistically significant when entered simultaneously. Age category was entered as an ordinal predictor (1 = ≤30, 2 = 31–40, 3 = ≥41). Table 5 presents the multiple linear regression. The overall model was significant, F(5, 119) = 2.53, *p* = 0.033, explaining 9.6% of variance in NKASRP scores (adjusted R^2^ = 0.058). Age remained the only significant independent predictor: each one-step increase in age category was associated with a 5.5-percentage-point higher NKASRP score (b = 0.055, 95% CI [0.016, 0.094], *p* = 0.006), holding experience, working area, education, and workshop attendance constant. Experience was non-significant (b = −0.018, *p* = 0.408) even without age in the model, indicating the age effect is not attributable to cumulative clinical exposure alone.

### 3.4. Perceived Causes of Delayed Pain Management

The most frequently reported delay was contacting a physician for opioid prescription (58.4%), followed by obtaining opioids from the pharmacy (46.4%) (Table 6). These findings represent nurses’ perceptions of delay points rather than objective time to analgesia measurements.

### 3.5. Perceived Barriers to Pain Management

Perceived barriers were distributed across medical staff-, patient-, and system-related domains (Table 7, Table 8 and Table 9). The most frequently perceived staff-related barrier was insufficient communication with patients (60.0%). Patient-related barriers centered on insufficient knowledge of pain control (52.8%) and communication gaps (51.2%). System-related barriers prominently included inadequate staffing (49.6%) and strict opioid regulation (46.4%).

## 4. Discussion

This study found a low composite NKASRP score among nurses working in a Saudi tertiary hospital, indicating persistent gaps in pain-related knowledge and attitudes. The overall mean score of 46.69% is far below expected competency levels and is consistent with the broader literature indicating that nurses across multiple settings continue to experience difficulty with pain assessment principles, opioid pharmacology, and pain-related clinical decision-making [1,2,8,9]. Rather than representing an isolated local anomaly, the present findings suggest that pain-related educational needs remain substantial even in settings where nurses routinely encounter pain assessment and documentation responsibilities.

The present findings align with prior Saudi and Gulf-region studies. AL-Sayaghi et al. reported that NKASRP scores among Saudi nurses ranged from 17.7% to 100%, with a mean of 45.29%, and the majority of participants (70.1%) had poor knowledge [21,22]. A more recent Riyadh-based study, Alanizi et al. reported moderate knowledge levels, with fewer than a quarter of participants demonstrating good performance [23]. In Qatar, Samara et al. documented a mean score of 48% among postoperative nurses [10] and in Oman, Al Zaabi et al. reported a mean NKASRP score of 49.6%, with significant associations between knowledge and education, experience with cancer patients, and prior pain training [11]. Together, these findings indicate that low pain-related knowledge-and-attitudes scores persist across regional settings and specialties, and this consistency may reflect enduring challenges in pre-service preparation, continuing education, and the translation of theoretical pain content into clinically applied decision-making.

The age finding is clinically noteworthy but requires cautious interpretation. In the present study, age was associated with higher scores and remained the only independent predictor in the multivariable model. However, years of experience and prior workshop attendance were not statistically significant, and the multivariable model explained only a modest proportion of the variance (R^2^ = 0.096). This pattern suggests that cumulative exposure or workshop attendance alone may not produce durable competence. A more plausible interpretation is that the quality, depth, and clinical relevance of training, combined with broader clinical exposure over time, may matter more than simple attendance. Similar findings were reported in the United Arab Emirates (UAE), where oncology nurses’ knowledge was low despite prior pain management education, suggesting that traditional educational approaches may need to be replaced with more effective, competency-based methods [24].

The item-level findings are particularly important because the poorest-performing items involved clinical reasoning when patient self-report was discordant with behavior, as well as knowledge of route selection and opioid-related decision-making. This pattern is clinically meaningful. If nurses underestimate patient-reported pain because a patient appears calm or is smiling, or if they remain uncertain about appropriate analgesic routes and opioid equivalence, undertreatment may occur even when pain has been assessed or documented. Similar knowledge gaps have been described in Saudi and international studies, especially in relation to pharmacological interventions, opioid misconceptions, and pain reassessment practices [13,22]. These deficits, therefore, appear to reflect a recurring educational weakness rather than a uniquely isolated feature of the present sample. The pattern is also consistent with systematic review evidence showing that pain assessment and management barriers in critical care include insufficient knowledge, opioid phobia, poor interdisciplinary communication, and limited structured training [25].

Although older nurses had higher mean scores, the analyses do not support strong causal inferences about experience over time. Because years of experience and workshop attendance were not statistically significant independent predictors, the present study cannot conclude that experience alone improves pain management competence. Instead, the findings suggest that structured mentorship, case-based learning, and supervised clinical reasoning support may warrant prospective evaluation [26]. Such strategies should be viewed as hypothesis-generating suggestions rather than direct conclusions established by the current cross-sectional data.

From the nurses’ perspective, opioid-related workflow processes were important contributors to delayed pain treatment. The most frequently endorsed delay was contacting a physician for opioid prescription, followed by obtaining opioids from the pharmacy. This broadly mirrors findings reported by Othman and Al-Atiyyat in oncology settings, where contacting the physician was also identified as a major delaying process [12]. The recurrence of this pattern across settings suggests that physician-gated prescribing and medication-access processes may represent meaningful perceived bottlenecks in pain care. At the same time, these findings should be interpreted cautiously because the present study did not measure objective administration timelines and cannot determine the actual duration or causal impact of these workflow steps. Documentation systems such as EHRs could potentially improve standardization and reduce delays, but whether they do so in practice depends on decision support design, workflow alignment, and institutional implementation, which were beyond the scope of the present study [16,17].

Perceived barriers to pain management were reported across staff, patient, and system domains. Insufficient communication with patients was the most frequently endorsed staff-related barrier, while patient knowledge deficits and communication gaps were also prominent. At the system level, inadequate staffing and opioid regulation were commonly reported. These findings are generally consistent with Saudi evidence reported by Maribbay et al., where staffing and opioid-related constraints were also perceived as important barriers [13]. They additionally resonate with broader nursing literature suggesting that institutional communication climate, role clarity, and organizational support influence how nurses navigate challenging practice situations [13]. Although Elseesy et al. examined error disclosure rather than pain management specifically, their findings on psychological and institutional barriers highlight a broader organizational context in which communication-related obstacles may affect nursing performance across different care domains [14]. Moreover, they reported that structural communication deficits in Saudi hospitals impede timely error disclosure, a finding we cite here for contextual support only, not as direct evidence on analgesic delays.

Communication emerged as a recurring theme at both staff and patient levels. Patient reluctance to report pain and reluctance to take opioids may reflect fear of addiction, side effects, stigma, prior experiences, or limited understanding of pain control. However, these variables were not directly measured from patients and should therefore be interpreted only as nurses’ perceptions. Even so, the prominence of communication-related barriers suggests that future interventions may need to extend beyond pharmacology-focused teaching and include communication skills, culturally responsive patient education, and structured approaches for discussing pain, analgesia, and expectations with patients and families [6,12].

Inadequate staffing was also frequently endorsed as a system barrier. This finding may indicate perceived workload pressure that reduces the time available for comprehensive assessment, timely reassessment, patient education, and interdisciplinary communication. Importantly, the present study did not objectively assess staffing ratios or organizational performance, so this result should not be interpreted as a direct audit of institutional staffing adequacy. Rather, it reflects nurses’ reports of constraints that may interfere with timely pain management in practice. Future research incorporating objective workflow and staffing indicators would help to clarify how these perceived barriers correspond to measurable care processes [11].

## 5. Recommendations for Future Research and Practice

Based on the present findings and their limitations, the following directions for future research and practice should be considered hypothesis-generating suggestions rather than direct conclusions from the current cross-sectional data.

Competency-based pain education: The consistently low NKASRP scores observed in this and prior regional studies suggest that attendance-based workshops may be insufficient to produce durable competence. Future interventions should evaluate competency-based approaches, including simulation-based training and case-based clinical reasoning exercises, targeting the specific knowledge gaps identified here, particularly clinical reasoning when self-report appears discordant with behavior, analgesic route selection, and opioid pharmacology [26].

Structured mentorship: Because age was the only independent predictor of NKASRP scores and years of experience were not significant, structured mentorship programs pairing less experienced nurses with higher-performing seniors may facilitate knowledge transfer and clinical reasoning development. The design, intensity, and outcomes of such programs should be tested prospectively [10].

Communication skills training: Insufficient communication with patients was the most frequent staff-related barrier, while patient reluctance to report pain and take opioids was a prominent patient-level barrier. Future education should therefore extend beyond pharmacology to include structured communication skills, culturally responsive patient education, and strategies for eliciting pain reports from reluctant patients [25].

Workflow and opioid access redesign: Contacting a physician for opioid prescription and obtaining opioids from the pharmacy were the most frequently reported delays, suggesting that physician-gated prescribing and medication-access pathways are meaningful bottlenecks. Future quality-improvement studies should evaluate structured prescribing protocols, clear escalation timelines, and pharmacy turnaround optimization, ideally with objective administration timestamps and patient-reported outcomes [12].

Multi-site and prospective designs: The single-center convenience sample limits generalizability. Future studies should employ multi-site designs across different Saudi regions and include objective indicators such as chart audit, measured time-to-analgesia, and patient-reported pain intensity and satisfaction to determine whether the observed knowledge gaps and barriers are generalizable and whether interventions produce measurable improvements [13,25].

System-level research: The organizational barriers identified here, including inadequate staffing and strict opioid regulation, may require institutional or policy-level responses. Future research should examine how staffing models, opioid access policies, and organizational culture interact to shape pain management outcomes [25].

## 6. Limitations

This study has several limitations. First, the single-center convenience sample limits external validity and generalizability to other Saudi settings. Second, the achieved sample of 125 participants was below the a priori target, which may have reduced statistical power for detecting small-to-moderate associations and increased the risk of type II error in non-significant subgroup analyses. Third, the data were self-reported and may have been influenced by recall or social desirability bias. Fourth, the study did not include direct chart audit, objective documentation quality indicators, or measured time to analgesia outcomes. Fifth, perceived barriers and delays were based on nurses’ reports and should not be interpreted as direct evaluations of institutional performance. These limitations should be considered when interpreting both significant and non-significant findings. Finally, it should be noted that Revacod (paracetamol/codeine) was substituted for Percocet (oxycodone/acetaminophen) in item 16 because Percocet is not routinely stocked in Saudi hospitals. Because codeine and oxycodone differ pharmacologically, item-level responses are not directly comparable with studies using the original NKASRP, and cross-study comparisons should be interpreted cautiously.

## 7. Conclusions

Nurses in this study demonstrated a low composite knowledge-and-attitudes score regarding pain assessment and management and reported several perceived communication, workflow, and system-level barriers relevant to timely analgesia. Age was associated with higher NKASRP scores, whereas other nurse characteristics were not independently predictive in the multivariable model. Because the study was cross-sectional and relied on self-report, the findings should be interpreted as associative rather than causal. Future multi-site and prospective studies are needed to test whether targeted educational and workflow-focused interventions can improve pain-related knowledge, communication, and analgesia timeliness in comparable settings.

## Figures and Tables

**Table 1:** Participant characteristics (N = 125).

Characteristic	Category	*n*	%
Gender	Male	6	4.8
	Female	119	95.2
Age group (years)	≤30	68	54.4
	31–40	39	31.2
	≥41	18	14.4
Years of experience	1–4	59	47.2
	5–9	23	18.4
	≥10	43	34.4
Education level	Diploma	20	16.0
	Bachelor	101	80.8
	Master	4	3.2
Attended pain course/workshop	Yes	86	68.8
	No	39	31.2
Documents pain after assessment	Yes	122	97.6
	No	3	2.4
Working area	Critical care	22	17.6
	Oncology	28	22.4
	Outpatient	16	12.8
	Emergency Department	3	2.4
	Medical	14	11.2
	Surgical	16	12.8
	Pediatric	17	13.6
	Others	9	7.2

**Table 2:** NKASRP knowledge distribution by item-level correct rate.

Score Level (Correct Response Rate)	Number of Questions
High (≥80%)	4
Medium (30–79%)	25
Low (<30%)	12

**Table 3:** Descriptive statistics of correct answers on NKASRP tool (N = 125).

No.	Question (Abbreviated)	Correct %
1	Vital signs reliable indicators	14.4%
2	Nervous system underdeveloped	48.0%
3	Distracted from pain	51.2%
4	Sleep despite severe pain	34.4%
5	Aspirin/NSAID agents	38.4%
6	Respiratory depression rare	59.2%
7	Combining analgesics	71.2%
8	Duration morphine analgesia	36.8%
9	Opioids not for history	27.2%
10	Elderly cannot tolerate opioids	59.2%
11	Endure as much pain	42.4%
12	Children <11 cannot report	51.2%
13	Spiritual beliefs affect pain	79.2%
14	Initial opioid dose timing	80.8%
15	Sterile water injection	54.4%
16	Revacod dosing	48.0%
17	Unknown pain source	29.6%
18	Anticonvulsant gabapentin	45.6%
19	Benzodiazepines not effective	65.6%
20	Opioid addiction definition	80.8%
21	Equianalgesia meaning	80.0%
22	Sedation assessment	81.6%
23	Recommended route (IM)	5.6%
24	Recommended route (oral)	69.6%
25	Analgesic medications	78.4%
26	30 mg oral morphine	36.0%
27	Postoperative pain	65.6%
28	Persistent cancer pain	23.2%
29	Likely reason patient pain	48.0%
30	Useful for treatment	42.4%
31	Most accurate judge	60.0%
32	Best description	52.0%
33	Likelihood opioid patients	29.6%
34	Peak effect morphine IV	75.2%
35	Peak effect morphine PO	55.2%
36	Abrupt opioid discontinuation	22.4%
37	Opioid statement true	28.8%
38	Patient A (Andrew smiling)	13.6%
39	Assessment smiling patient	3.2%
40	Patient B (Robert)	16.0%
41	Assessment grimacing patient	10.4%

**Table 4:** Association between participant characteristics and NKASRP score (%).

Variable	Category	*n*	Mean (SD)	Test Statistic	*p*-Value
Gender	Male	6	45.12 (8.69)	t = −0.398	0.692
	Female	119	46.77 (9.98)		
Age group (years)	≤30	68	44.30 (9.63)	F = 5.136	0.007 *
	31–40	39	48.72 (9.45)		
	≥41	18	51.37 (9.70)		
Years of experience	1–4	59	45.56 (9.35)	F = 0.275	0.760
	5–9	23	45.07 (10.63)		
	≥10	43	49.13 (10.01)		
Education level	Diploma	20	47.92 (9.76)	F = 0.295	0.745
	Bachelor	101	46.36 (10.30)		
	Master	4	48.78 (8.44)		
Attended pain course/workshop	Yes	86	47.37 (8.70)	t = 1.126	0.262
	No	39	45.21 (10.30)		
Documents pain after assessment	Yes	122	46.90 (9.90)	t = 1.510	0.134
	No	3	38.21 (6.10)		

* *p* < 0.05.

**Table 5:** Multiple linear regression: NKASRP total score on nurse characteristics (n = 125).

Predictor	Coding	b	SE	t	*p*	95% CI
Intercept	—	0.4022	0.0611	6.58	<0.001	[0.281, 0.523]
Age group	Ordinal 1–3	0.0551	0.0197	2.79	0.006	[0.016, 0.094]
Experience	Ordinal 1–3	−0.0182	0.0219	−0.83	0.408	[−0.062, 0.025]
Working area	Categorical	0.0028	0.0038	0.74	0.462	[−0.005, 0.010]
Education	Ordinal 1–4	0.0048	0.0143	0.33	0.740	[−0.024, 0.033]
Workshop	Binary 1–2	−0.0116	0.0186	−0.62	0.535	[−0.048, 0.025]

Model note: F(5, 119) = 2.53, *p* = 0.033; R^2^ = 0.096; Adj R^2^ = 0.058; SE_reg_ = 0.099; VIF all < 2.5.

**Table 6:** Perceived causes of delayed pain management (N = 125).

Cause	Frequency	%
Administering opioid for the patient	15	12.0
Obtaining opioid from the pharmacy	58	46.4
Contacting physician for prescription of opioid	73	58.4
The delaying process is difficult to recognize	28	22.4

**Table 7:** Medical staff-related barriers (N = 125).

Barrier	Frequency	%
Inadequate pain assessment	48	38.4
Inadequate experience on pain control	35	28.0
Insufficient knowledge of pain control	39	31.2
Insufficient communication with patient	75	60.0
Reluctance to prescribe opioids	50	40.0

**Table 8:** Patient-related barriers (N = 125).

Barrier	Frequency	%
Reluctance to report pain	62	49.6
Reluctance to take opioid	37	29.6
Insufficient communication with staff	64	51.2
Financial constraints	8	6.4
Insufficient knowledge of pain control	66	52.8

**Table 9:** Healthcare system-related barriers (N = 125).

Barrier	Frequency	%
Strict regulation of opioids	58	46.4
Inadequate staffing	62	49.6
Limited stock of different types of opioids	41	32.8
Pain management is not considered as important	23	18.4
Medication and intervention costs	27	21.6

## Data Availability

The data presented in this study are available upon reasonable request from the corresponding author.

## Public Involvement Statement

No public involvement in any aspect of this research.

## Guidelines and Standards Statement

This manuscript was drafted against the STROBE checklist for cross-sectional studies.
